# Prevalence of Oral Manifestations and Their Association with CD4/CD8 Ratio and HIV Viral Load in South India

**DOI:** 10.1155/2011/964278

**Published:** 2011-10-20

**Authors:** Sharma Gaurav, Pai M. Keerthilatha, Nagpal Archna

**Affiliations:** ^1^Department of Oral Medicine and Radiology, Sudha Rustagi Dental College, Haryana, Faridabad 121002, India; ^2^Department of Oral Medicine and Radiology, Manipal College of Dental Sciences, Manipal 576104, India; ^3^Department of Oral Medicine and Radiology, PDM Dental College, Haryana, Bahadurgarh 124507, India

## Abstract

The objective of the present research was to determine the prevalence of oral manifestations in an HIV infected population from south India and evaluate their association with HIV viral load and CD4/CD8 ratio. Intraoral examination of 103 patients, whose CD4/CD8 ratio was available, were conducted. HIV viral loads were available for thirty patients only. The prevalence of oral manifestations was 80.6% (83/103). The most common oromucosal lesion was erythematous candidiasis (EC) (38.8%) followed by melanotic hyperpigmentation (35.9%). Patients having any oral manifestation had a mean CD4/CD8 ratio of 0.24. EC had positive predictive value of 85.0% for CD4/CD8 ratio <0.30. The prevalence of oral manifestations in patients taking ART was lesser (78.6%) as compared to patients not taking ART (82%). Patients having any oral manifestation had a higher HIV viral load as compared to patients not having any oral manifestations (*P* < 0.05). Pseudomembranous candidiasis (PC) was significantly associated with higher HIV viral loads (>20,000 copies/mL) (*P* < 0.05). Patients having EC had 4 times greater chance of having CD4/CD8 ratio <0.30. PC can be considered as a marker of immune suppression (HIV viral load >20,000 copies/mL).

## 1. Introduction

1.8 million people died in 2009 due to AIDS-related causes, and an estimated 2.6 million people were newly infected with HIV in world [1]. India carries the third largest number of HIV infected patients in the world after South Africa and Nigeria [1]. HIV infection causes depletion of CD4 cells in peripheral blood and lymphoid tissue causing CD8 cell dysfunction [2]. Quantitation of CD4 helper lymphocytes is thus essential in the staging and monitoring of patients infected with HIV. Throughout the course of disease, the total T-cell levels remain fairly constant despite a fall in CD4 cell count due to compensatory rise in CD8 cells. Absolute CD4 counts are known to be inherently inconsistent and therefore could be misleading [3]. Therefore the ratio of CD4 cell to CD8 cells is a significant measure which is of greater magnitude as compared to absolute CD4 cell count of disease progression in HIV-infected subjects [3]. Plasma HIV-1 RNA levels have been shown to be a strong predictor of rapid progression to AIDS after seroconversion that is independent of CD4^+^ counts [4]. High viral load is currently considered to be one of the main indicators of HIV-induced immune deterioration.

Since the onset of HIV/AIDS epidemic, the oral cavity has played a key role in helping to define the natural history of HIV/AIDS. The occurrence of oral manifestations is favored by immune deterioration. Few studies have evaluated the relationship of CD4/CD8 ratio with oral manifestations [5–8]. High CD8 lymphocytosis and low CD4/CD8 ratio have also been associated with oral candidiasis (OC) [5, 7]. Studies from western countries have also shown association of oral manifestations with higher HIV viral load [9–12]. However till date no Asian study has attempted to assess the relationship between oral manifestations with CD4/CD8 ratio and HIV viral load. The possible reasons for absence of studies related to HIV viral load conducted in Asia could be financial and resource constraints. 

In the present study, a group of HIV patients from south Indian population were analyzed to identify possible association of oral manifestations with CD4/CD8 count ratio and HIV viral load and to evaluate the diagnostic utility of correlating main oral manifestations for low CD4/CD8 ratios (<0.30) using positive predictive value (PPV), negative predictive value (NPV).

## 2. Subjects and Methods

The cross-sectional study was conducted over 1-year period between January 2005 and December 2005 under the approval of IREC (institutional research ethical committee). Written informed consents were obtained from participants (patients). One hundred and three HIV positive patients were screened and were included in study. Their respective CD4/CD8 ratio was available within 2 weeks of oral examination. All 103 patients were diagnosed as HIV-antibody positive by enzyme-linked immunosorbent assay (ELISA)-HIV. Three separate positive ELISA tests were considered confirmatory. The patients were selected from our patient department of infectious diseases unit at Attavar hospital, Mangalore and hospitalized patients of Kasturba hospital, Manipal. Sociodemographic data were obtained using structured questionnaire. A patient was considered as smoker if there was past or current history of smoking of at least 1 cigarette/beedi a day for at least 1 year. Similarly an assessment of consumption of alcohol (any type) was also done by asking patient about his/her current consumption of alcohol (more than 30 grams) at least once a week for 1 year. 

Clinical history was obtained from patient's medical records. Absolute CD4^+^ and absolute CD8 lymphocyte counts were performed using flow cytometry (SRL, Ranbaxy laboratories, Mumbai) within 2 week of the oral examination. Patients according to CD4/CD8 ratio were clustered into 3 groups: group I (0.01–0.30), group II (0.31–0.60), and group III (>0.60). HIV viral load was also documented from 30 patients among the study sample of 103 patients and was subsequently constellated in 2 groups (<20,000 copies/mL and >20,000 copies/mL). Oral manifestations were diagnosed according to presumptive criteria of EEC-clearinghouse classification (1993) [13]. A single examiner trained in oral medicine (G.S.) examined and recorded all oromucosal lesions.

## 3. Statistical Analysis

Statistical analysis was done using SPSS software version 11. Associations between subject variable and each type of oral lesion were analyzed using chi-square test. Odds ratio and 95% confidence interval were used in logistic regression analysis for association between oral manifestations and CD4/CD8 ratio within group I. Mann-Whitney *U* test was used for differences between mean values of group of patients. PPV and NPV of individual oral manifestations for low CD4^+^/CD8 ratio were calculated. The probability that the patient has CD4/CD8 ratio <0.30 when a specific oral manifestation is present is called as positive predictive value (PPV). Negative predictive value (NPV) is reported as the possibility that the patient's CD4/CD8 ratio >0.30 when a specific oral manifestation is absent. Statistical significance was set at *P* ≤ 0.05.

## 4. Results

The demographic distribution of 103 patients (age range 24–68 years; mean age 37.1 years; 70 males, 33 females) consisted of all south Indians. Social demographics, their oral habits, usage of antiretroviral therapy and laboratory parameters of the study cohort can be observed in Table 1. Among the study population 101 patients (98.3%) had CD4/CD8 ratio less than 1 (normal range 0.03–1.61) which is suggestive of an advanced immunosuppression in this study (*P*  value < .05; chi-square test). 32 (31.1%) patients were smokers and 48 (46.6%) patients were alcoholics. 

The mean absolute CD4 lymphocyte count among low CD4/CD8 ratio (group I) was 163.43 cells/mm^3^ (S.D 143.59), while patients within group II had mean value of 325.00 cells/mm^3^ (S.D 167.46) and patients within group III had 502.33 cells/mm^3^ (S.D 290). When comparing CD4 lymphocyte counts to CD4/CD8 ratio among different groups, low-ratio patients had significant differences from those patients who had higher ratios (*P* < .001). Mean CD4/CD8 ratio of entire study cohort was found to be 0.26 {standard  deviation  (S.D)—0.22}. The mean absolute CD4 and CD8 counts of study cohort were 228.82 and 988.6 cells/mm^3^, respectively. Higher HIV viral load >20,000 copies/mL was seen in patients below CD4/CD8 ratio <0.30. (Table 2).

Oral manifestations were present in 83 (80.6%) patients. Oral manifestations were also clustered according to varying CD4/CD8 ratios corresponding, respectively, to groups I (0.01–0.30), II (0.31–0.60), and III (>0.60). Erythematous candidiasis (EC) (49.3%), melanotic hyperpigmentation (40.6%), and xerostomia (37.7%) were common oral findings within group I, whereas within group II melanotic hyperpigmentation (28.6%) followed by EC (17.9%) were more frequent oral manifestations. Overall, EC (38.8%) was most common finding followed by melanotic hyperpigmentation (35.9%), xerostomia (32.0%), oral hairy leukoplakia (OHL) (17.5%), and linear gingival erythema (LGE) (Table 3).

Current mean CD4/CD8 ratios for groups with and without oral manifestations are given in Table 4. The mean absolute CD4 count for the study cohort with presence of any oral manifestation was 196.18 cells/mm^3^, and mean CD4/CD8 ratio for the study cohort was 0.24 (S.D 0.22). The lowest mean CD4/CD8 ratio of 0.16 among individual oral manifestations was found for pseudomembranous candidiasis (PC) (mean absolute CD4 count—126.78 cells/mm^3^). Mann-Whitney *U* test revealed a statistically significant difference between mean values of group when any oral manifestation was present (*P* < 0.05) as compared to when oral manifestations were absent. Among individual oral manifestations highly statistical significant differences were found for EC and PC [*P* < 0.05]. No statistically significant differences were found for xerostomia, hyperpigmentation, and OHL. 

PPV and NPV for association of specific oral manifestations when compared with CD4/CD8 ratio within group I were calculated (Table 5). The PPVs for EC, PC, OHL and melanotic hyperpigmentation were 85%, 77.8%, 77.8%, and 75.7%, respectively. The NPVs for EC, PC, OHL, and melanotic hyperpigmentation were 44.4%, 34.3%, 35.3%, and 37.9%, respectively. Multivariate regression analysis was done to show odds ratio and 95% confidence interval for all oral manifestations with low CD4/CD8 ratios (<0.30). EC had a high odds ratio of 4.530 for low CD4/CD8 ratio. For other oral manifestations, no statistical significance could be reached (Table 5).

Subdistribution of patients (30/103) according to clustering of HIV viral loads is given in Table 6. The most common oral manifestation among the HIV viral load subpopulation (*n* − 30) was EC (40.0%), xerostomia (30.0%), and melanotic hyperpigmentation (26.6%). 16 Patients had HIV viral load >20,000 copies/mL and remaining had HIV viral load <20,000 copies/mL. All the patients (16/16; 100%) having HIV viral load >20,000 copies/mL had some type of oral manifestation (statistically significant; *P* < 0.05). Among individual oral manifestations, PC was seen more frequently in patients with viral loads greater than 20,000 copies/mL (*P* < 0.05).

Table 7 gives the comparison of oral manifestations between patients on ART (*n* − 42) and patients not on ART (*n* − 61). A reduced prevalence of oral manifestations in patients on ART (33/42; 78.6%) was observed as compared to those patients not on ART (50/61; 82%). The prevalence of melanotic hyperpigmentation was 40.5% followed by EC (35.7%), xerostomia (28.6%), angular cheilitis (11.9%), OHL (9.5%), and PC (7.1%) in patients on ART. No statistical significance could be observed for any individual oral manifestations for patients on ART.

## 5. Discussion

Since the beginning of AIDS epidemic, developing countries have experienced difficulties in implementing appropriate, inexpensive, and efficient HIV laboratory diagnostic techniques to aid in the epidemiological assessment and control of HIV infection. Asian and the African countries together house 95% of global HIV burden. Studies have been conducted in Asia for association of oral manifestations with absolute CD4 lymphocyte counts [14–16]. However, on doing a comprehensive literature search we could not find any Asian study for association of oral manifestations with CD4/CD8 ratio and HIV viral load. Hence an attempt was made in this study for correlation of oral manifestations with HIV viral load and CD4/CD8 ratio for immune suppression. 

The prevalence of oral manifestation in our study was 80.6%. This finding was similar to studies conducted in Venezuela (85%) [9], Thailand (77.0%) [16], and Nigeria (84.0%) [17]. In another Indian study the prevalence of oral manifestations were seen in 72.3% patients [15]. 82.9% (58/70) males had oral manifestations as compared to 75.8% (25/33) females (Table 1). No statistically significant differences were seen in oral manifestations in either gender. This finding is similar to other studies [15, 18] where males had more oral manifestations. Oral manifestations were seen more in smokers (90.6%; 29/32) as compared to oral manifestations in nonsmokers (76.1%; 54/71), but without statistical significance (*P* > .05). Smoking may produce particulate matter, noxious chemicals, and heat that can modify more local factors. Oral manifestations were equally prevalent in alcoholics (81.3%; 39/48) as well as nonalcoholics (80%; 44/55). No statistical significance was also observed for individual oral manifestations with gender, smoking and alcohol (*P* > .05). 

Since myriad factors can affect T-cell subsets, changes in CD4^+^ T cells and CD8^+^ T cells reflected through CD4/CD8 ratio should be investigated over a time frame, preferably a 3–6 month period to assess the immune status of patient. The clinical significance of CD4/CD8 ratio and HIV viral load are well established for HIV disease progression, but as to date, no consensus has been reached in scientific literature as to what comprises the standard grouping for CD4/CD8 ratios and HIV viral load.

The prevalence of EC was 38.8% (Table 3) that was comparable to studies conducted by Campo et al. 2002 (37.8%) [10] and Nittayananta et al. (40%) [18]. Among candidal infections the most common finding was EC (38.8%) followed by PC (8.7%), angular cheilitis (AC) (5.8%), and hyperplastic candidiasis (1.9%). Prognostic significance for both EC and PC has been reported to be similar by two longitudinal studies by Dodd et al. 1991 and Nielsen et al. 1994 [19, 20]. Fonseca et al. 2000 correlated oral manifestations with CD4/CD8 ratio in 51 HIV positive children and concluded that patients with CD4/CD8 ratio <0.5 to be more susceptible to PC and EC [21]. 

A higher frequency for EC was found in our patients within group I (49.3%), as compared to group II (17.9%) and group III (16.7%). This difference was found to be statistically significant (*P* < 0.05; chi-square test of association). The decreasing prevalence of EC with lower CD4/CD8 ratio suggests deteriorating immune system and accelerated destruction of lymphocytes [9]. EC has been found to occur more frequently in patients with low CD4/CD8 ratio [5, 22, 23]. However, for other oral manifestations no significant differences were observed.

Mean values of CD4/CD8 ratio for EC were 0.18 which was lower than 0.40 in another study [5] that also concluded that when CD4/CD8 ratio is less than 0.5 in patients with EC, it appears to be of prognostic significance as 80% of patients developed AIDS at a median of 3 months. This finding suggests the magnitude of utility of diagnosis of EC. However, this conclusion can be confirmed after longitudinal studies of large sample size are conducted and use of stringent definitive criteria as there is a concern for potential misdiagnosis in following presumptive criteria. However due to lack of resources and practical feasibility, epidemiological studies throughout the world have followed presumptive criteria for diagnosis of oral manifestations in HIV/AIDS infected population.

The mean CD4/CD8 ratio for any oral manifestation was 0.24 ± 0.23 which signifies that presence of any single oral manifestation increases probability (PPV—73.5%) of a patient having lowered immune status as compared to patients not having any oral manifestation (Table 4). Difference of mean values was also significantly different (*P* < 0.05). This emphasizes the need for careful oral examination as oral manifestations can act as an indicator for immune suppression, especially in resources deficient clinical settings and also in probable diagnosis of new cases of HIV. An early and timely diagnosis will help in improved treatment planning and prognosis of patients. 

PPVs for oral manifestations to low CD4/CD8 ratio <0.30 were in a high range from 75.7% for melanotic hyperpigmentation to 85.7% for linear gingival erythema (LGE). Multivariate regression analysis showed that patients with EC had 4 times greater chance of having CD4/CD8 ratio <0.30 {OR—4.728 (1.816–12.313); *P* = 0.001  and  OR—4.530  (1.667—12.323); *P* = 0.003} as compared to patients with absence of EC. No other statistically significant findings for oral manifestations were found (Table 5). This finding is significant as EC can be considered as a clinical marker of AIDS for CD4/CD8 ratio <0.30 and hence ART can be instituted. 

Oral hairy leukoplakia (OHL) was observed in 18 (17.5%) patients. This figure is slightly higher to studies conducted by Laurenco and Figueiredo (11.8%) and Pedreira et al. (9%) [24, 25]. Reichart et al. [6] found the mean CD4/CD8 ratio as 0.24 with range 0.04–1.0, which is almost similar to our study (0.26). However, we could not find any statistically significant differences for OHL with low CD4/CD8 ratio. There is no consensus for relationship of OHL with low absolute CD4^+^ counts as conflicting results of its association have been found [9, 11]. More studies need to be done, especially in Asian and African countries, to confirm whether OHL can be associated with low CD4/CD8 ratio.

HIV viral load is known to have better predictive value than absolute CD4 cell count of long-term clinical outcome while CD4^+^ lymphocyte count is more useful for short-term clinical outcome [26, 27]. Patients with the oral manifestations had a greater HIV viral load (*P* < 0.05), a finding corroborated in other studies also [9, 10, 12] (Table 5). However, Gaitan-Cepeda et al. [28] reported no relationship between viral load and presence of oral lesions. The mean HIV viral load of patients having oral manifestations found in our study was 1,69,035 copies/mL which was higher as compared to other similar studies conducted in Brazil {12,300 copies/mm^3^} [24], Italy {17,900 copies/mL} [12], and South Africa {72,200 copies/mL} [29], but much lesser than a study conducted in Venezuela [9] where the mean count was 15,102,00 copies/mm^3^. These variations of mean count for oral manifestations can be explained by variations in sample size, ethnicity, use of ART, gender and age distribution, and oral adverse habits. 

EC was the most common finding in subpopulation with HIV viral load with RNA copies greater than 20,000 copies/mL. A statistically significant association was observed between PC and high viral load (>20,000 copies/mL) (Table 6). Campo et al. [10], Margiotta et al. [12], and Laurenco and Figueiredo [24] had also found a positive correlation between high HIV viral loads >10,000 copies/mL and oral candidiasis. In our study no correlation could be found between OHL and high viral loads. This was in contrast to other studies where OHL was associated to high HIV viral load [9, 24, 30]. Patton et al. found that patients with OHL were twofold more likely to have a higher viral load than people without oral lesions [30], whereas Moura et al. had suggested OHL as a clinical marker for HIV viral load >3000 copies/*μ*L [31]. Our finding of negative correlation for OHL should be explored in future studies whether there is any difference in relationship between OHL and high viral loads in developing and developed nations. 

The prevalence of oral manifestations of those patients on antiretroviral therapy (ART) was comparatively lesser as compared to patients not on ART (78.6% versus 82.0%; *P* = .669), though difference was not statistically significant (Table 7). This finding was in concurrent with other studies [32–34]. Among all oral manifestations, only melanotic hyperpigmentation had a slightly higher prevalence in patients on ART. This finding is similar to other studies conducted in India [15, 34]. Various reasons that have been attributed to increased oral pigmentation in patients with ART are increased release of MSH caused by deregulation of cytokines in HIV disease, smoking, Addison's disease, and use of melanocyte stimulating drugs like ART [34].

OHL can also be considered as a measure of efficacy of ART [12, 16]. There was a regressive, but not statistically significant, effect of ART on OHL. Various studies have documented a reduced prevalence of OHL in patients who are taking ART [16, 31, 35]. This can be attributed to antiviral effect of ART. The Protease inhibitors, a class of ART, are also known to exert anticandidal activity by inhibiting secreted aspartyl proteinases [SAPs] secreted by *candida* species. SAPs are the major virulence factors for *candida* species and their inhibition would mean a reduced virulence and hence decreased prevalence of oral candidiasis that has been reported in various studies [36, 37]. The absence of Protease inhibitors in our study sample could be the probable reason why we could not observe a dramatic reduction in EC and PC.

No oral warts were observed in our study. This finding was similar to other studies conducted in Asia [14–16, 34]. The possible reason could be a functionally incomplete reconstitution that might lead to development of human papilloma virus-(HPV-) induced lesions like warts, condylomas, and focal epithelial hyperplasia [38]. The probable enhanced antiviral effect of ART on HPV related to genetics as well as the virulence factors could be the reasons for absence of warts in Asian countries.

The presence of oral manifestations in patients on ART may indicate advanced immune suppression or failure of the therapy [34, 36]. Among subsample of HIV viral load population (*n* − 30), 15 patients (50%) were on ART and they had HIV viral load <20,000 copies/mL (*P* < 0.05). The low number of patients with HIV viral loads on ART (*n* − 15) prevented the authors from doing meaningful statistical analysis with individual oral manifestations. In our study we could not find any oral manifestation that could possibly show signs of ART failure (*P* > .05). A greater sample size of patients on ART could be directed towards future research for its association with oral manifestations.

HIV infection remains the deadliest disaster for humanity. Unfortunately, no effective vaccine has been found so far, and there is little hope that it will be developed in near future which can combat the virus or at least stimulate the host immune system. In this study, we found a significant association of CD4/CD8 ratio with EC but not OHL. More studies are needed to carry out association between oral manifestations and CD4/CD8 ratio, which is a better laboratory marker than absolute CD4 count for immune suppression. So far, studies have been conducted only in western countries with almost scarcity of literature in developing world. Studies similar to ours need to be done with cohorts comprising of samples of resource-constrained nations, which can be compared with those from developed nations to evaluate if there are differences. Future studies could be directed towards association between oral manifestations and HIV viral load, a better marker of long-term immune deterioration. A greater sample study size for HIV viral load in developing nations will be required to confirm the findings in our study.

People infected with HIV need to be educated, inspired, and empowered with knowledge so that they can protect themselves from the impact of AIDS. To ensure a better care and quality of life to these patients linkages with social sector programmes for accessing social support to the patients, recreational and skill development support, transport subsidy to ensure ART care, and integrating nutritional support within treatment regimens are certain strategies that can be implemented. By regular intraoral examinations at frequent intervals dentists can play a small but significant role of ensuring an effective integrated delivery of health services for HIV-infected patients. This can occur only if there is joint action/strategy between health care providers and various HIV-related government as well as nongovernment organizations. 

The possibility of an oral lesion index to predict HIV in resource poor countries with no access to CD4 count or viral load was suggested recently [39]. However to incorporate an oral lesion index, certain regional parameters and research studies that employ rigorous research methodologies with emphasis on HIV viral load and CD4/CD8 ratio need to be done. There is a need to compare the results of various cross-sectional studies, and more longitudinal studies are required in developing nations to formulate an oral lesion index that can help as a marker for HIV-related immune suppression. 

EC had good predictive values (84.5% and 85%) for group I CD4/CD8 ratio <0.30. OHL is significantly not associated with low CD4/CD8 ratio. Patients having EC were at 4 times risk of having CD4/CD8 ratio <0.30 as compared to patients with absence of EC. PC was significantly seen more frequently in patients with high viral load >20,000 copies/mL. There was reduced prevalence of oral manifestations in patients on ART. More studies need to be carried out, especially in Asia, for correlation of oral manifestations with HIV viral load and CD4/CD8 ratio.

## Figures and Tables

**Table 1:** Demographics and laboratory parameters of study cohort.

Variable	Total no. of patients (*n* − 103)	No. of patients with oral manifestations (83/103)
Gender		
Male	70	58 (82.9%)
Female	33	25 (75.8%)
Mode of transmission		
Heterosexual mode	101	81 (80.1%)
Homosexual mode	2	2 (100%)
Antiretroviral therapy		
Yes	42	33 (78.6%)
No	61	50 (82.0%)
Smoker		
Yes	32	29 (90.6%)
No	71	54 (76.1%)
Alcohol consumption		
Yes	48	39 (81.3%)
No	55	44 (80%)
Absolute CD4/CD8 ratio staging		
Group I (0.01–0.30)	69	61 (88.4%)
Group II (0.31–0.60)	28	18 (64.2%)
Group III (>0.60)	6	04 (66.7%)

**Table 2:** Absolute mean CD4 and CD8 counts along with mean CD4/CD8 ratio of study cohort.

Clustering of patients according to CD4/CD8 ratio	Mean absolute CD4 count (cells/mm^3^)	Mean absolute CD8 count (cells/mm^3^)	Mean CD4/CD8 ratio
Group I (0.01–0.30) (*n* − 69)	163.43	1053.1	0.15
Group II (0.31–0.60) (*n*−28)	325.00	889.1	0.39
Group III (>0.60) (*n* − 6)	502.33	659.8	0.93
Total no. of patients (*n* − 103)	228.82	988.6	0.26

**Table 3:** Oral manifestations in relation to CD4/CD8 ratio.

Oral manifestations	Group I	Group II	Group III	Total
	*n* − 69	*n* − 28	*n* − 6	(*n* − 103)
Any oral manifestation*	61 (88.4%)	18 (64.3%)	4 (66.7%)	83 (80.6%)
Erythematous candidiasis (EC)	34 (49.3%)	5 (17.9%)	1 (16.7%)	40 (38.8%)
Pseudomembranous candidiasis (PC)	7 (10.1%)	2 (7.1%)	—	9 (8.7%)
Hyperpigmentation	28 (40.6%)	8 (28.6%)	1 (16.7%)	37 (35.9%)
Oral hairy leukoplakia (OHL)	14 (20.3%)	3 (10.7%)	1 (16.7%)	18 (17.5%)
Necrotizing ulcerative gingivitis (NUG)	8 (11.6%)	2 (7.1%)	—	10 (9.7%)
Necrotizing ulcerative periodontitis (NUP)	3 (4.3%)	—	1 (16.7%)	4 (3.9%)
Linear gingival erythema (LGE)	12 (17.4%)	2 (7.4%)	—	14 (13.6%)
Xerostomia	26 (37.7%)	5 (17.9%)	2 (33.3%)	33 (32.0%)
Angular cheilitis (AC)	6 (8.7%)	—	—	6 (5.8%)
Apthous ulcers	4 (5.7%)	1 (3.6%)	—	5 (4.9%)
Exfoliative cheilitis	2 (2.8%)	—	—	2 (1.9%)
Hyperplastic candidiasis	1 (1.4%)	1 (3.6%)	—	2 (1.9%)
Herpes zoster	1 (1.4%)	1 (3.6%)	—	2 (1.9%)

*Many patients had more than one oral manifestation.

**Table 4:** Mean CD4/CD8 ratio and their association with presence or absence of oral manifestations.

Oral manifestations (OM)	Mean absolute CD4 count when respective OM present (cells/mm^3^)	Mean CD4/CD8 ratio of group when respective OM present	Mean CD4/CD8 ratio of group when respective OM absent	*Z*	*P* value
Any oral manifestation	196.18	0.24 (S.D—0.23)	0.35 (S.D—0.17)	3.2	.01^a^
EC	147.18	0.18 (S.D—0.12)	0.32 (S.D—0.26)	3.73	.00^a^
PC	126.78	0.16 (S.D—0.15)	0.27 (S.D—0.23)	1.8	.05^a^
OHL	149.39	0.26 (S.D—0.23)	0.26 (S.D—0.22)	.08	.93^b^
Hyperpigmentation	192.27	0.23 (S.D—0.26)	0.28 (S.D—0.20)	1.7	.08^b^
Xerostomia	195.7	0.22 (S.D—0.17)	0.29 (S.D—0.24)	1.8	.07^b^

S.D: Standard deviation.

^a^Statistically significant.

^b^Not statistically significant.

**Table 5:** Predictive values of oral manifestations for CD4/CD8 ratio <0.30.

Oral manifestation	PPV	NPV	Odds ratio	95% CI	*P* value
EC	85.0%	44.4%	4.53	1.66–12.32	.003^a^
OHL	77.8%	35.3%	1.90	0.57–6.31	.290^b^
Hyperpigmentation	75.7%	37.9%	1.98	0.77–4.66	.164^b^
Xerostomia	78.8%	38.6%	2.33	0.89–6.11	.085^b^
NUG	80.0%	34.4%	.477	0.09–2.37	.366^b^
PC	77.8%	34.0%	1.80	0.35–9.91	.477^b^
LGE	85.7%	36.0%	3.36	0.70–16.00	.127^b^

PPV: Positive predictive value, NPV: Negative predictive value.

^a^Statistically significant.

^b^Not statistically significant.

**Table 6:** Comparison of oral manifestations with respect to HIV viral load subpopulation.

Oral manifestations	HIV viral load <20,000 copies/mL	HIV viral load >20,000 copies/mL	Total	*P* value
	*n* − 14	*n* − 16	*n* − 30	
Any oral manifestation	9 (64.3%)	16 (100%)	25 (83.3%)	.00^a^
EC	2 (14.2%)	10 (62.5%)	12 (40.0%)	.07^b^
Hyperpigmentation	3 (21.4%)	5 (31.2%)	8 (26.6%)	.54^b^
Xerostomia	2 (14.2%)	7 (43.8%)	9 (30.0%)	.07^b^
OHL	2 (14.2%)	2 (12.5%)	4 (13.3%)	.88^b^
PC	—	5 (31.2%)	5 (16.6%)	.02^a^
AC	—	2 (12.5%)	2 (6.6%)	—
LGE	2 (14.2%)	—	2 (6.6%)	—
NUG	—	2 (12.5%)	2 (6.6%)	—
Hyperplastic candidiasis	—	1 (6.2%)	1 (3.3%)	—

^a^Statistically significant.

^b^Not statistically significant.

**Table 7:** Comparison of oral manifestations with respect to antiretroviral therapy.

Oral manifestation (OM)	Patients with OM on antiretroviral therapy (ART)	Patients with OM *not *on ART	*P* value
	*n* − 42	*n* − 61	
Any oral manifestation	33 (78.6%)	50 (82%)	.66^a^
EC	15 (35.7%)	25 (41.0%)	.59^a^
OHL	4 (9.5%)	14 (23.0%)	.07^a^
AC	2 (4.8%)	4 (6.6%)	.70^a^
PC	3 (7.1%)	6 (9.8%)	.63^a^
Hyperpigmentation	17 (40.5%)	20 (32.8%)	.42^a^
Xerostomia	12 (28.6%)	21 (34.4%)	.53^a^
LGE	5 (11.9%)	9 (14.8%)	.67^a^
NUG	5 (11.9%)	5 (8.2%)	.53^a^
NUP	1 (2.4%)	3 (4.9%)	.51^a^

^a^Not statistically significant.

## References

[2] Levy JA (2009). HIV pathogenesis: 25 years of progress and persistent challenges. *AIDS*.

[3] Forbi JC, Agwale SM (2009). Inverted CD4+/CD8+ ratio associated with AIDS event and death in HIV-1 infected individuals in Nasarawa State, Nigeria. *Tanzania journal of health research*.

[4] Mellors JW, Rinaldo CR, Gupta P, White RM, Todd JA, Kingsley LA (1996). Prognosis in HIV-1 infection predicted by the quantity of virus in plasma. *Science*.

[5] Klein RS, Harris CA, Small CB, Moll B, Lesser M, Friedland GH (1984). Oral candidiasis in high-risk patients as the initial manifestation of the acquired immunodeficiency syndrome. *New England Journal of Medicine*.

[6] Reichart PA, Langford A, Gelderblom HR, Pohle HD, Becker J, Wolf H (1989). Oral hairy leukoplakia: observations in 95 cases and review of the literature. *Journal of Oral Pathology and Medicine*.

[7] Hilton JF, Donegan E, Katz MH (1997). Development of oral lesions in human immunodeficiency virus-infected transfusion recipients and hemophiliacs. *American Journal of Epidemiology*.

[8] Steidley KE, Thompson SH, McQuade MJ, Strong SL, Scheidt MJ, Van Dyke TE (1992). A comparison of T4:T8 lymphocyte ratio in the periodontal lesion of healthy and HIV-positive patients. *Journal of Periodontology*.

[9] Bravo IM, Correnti M, Escalona L (2006). Prevalence of oral lesions in HIV patients related to CD4 cell count and viral load in a Venezuelan population. *Medicina Oral, Patología Oral y Cirugía Bucal*.

[10] Campo J, Del Romero J, Castilla J, García S, Rodríguez C, Bascones A (2002). Oral candidiasis as a clinical marker related to viral load, CD4 lymphocyte count and CD4 lymphocyte percentage in HIV-infected patients. *Journal of Oral Pathology and Medicine*.

[11] Greenspan D, Gange SJ, Phelan JA (2004). Incidence of oral lesions in HIV-1-infected women: reduction with HAART. *Journal of Dental Research*.

[12] Margiotta V, Campisi G, Mancuso S, Accurso V, Abbadessa V, Giaccone P (1999). HIV infection: oral lesions, CD4+ cell count and viral load in an italian study population. *Journal of Oral Pathology and Medicine*.

[13] (1993). EEC-Clearinghouse on oral problems related to HIV infection and WHO collaborating Centre on oral manifestations of the human immunodeficiency virus. Classification and diagnostic criteria for oral lesions in HIV infection. *Journal of Oral Pathology and Medicine*.

[14] Tsang PCS, Samaranayake LP (1999). Oral manifestations of HIV infection in a group of predominantly ethnic Chinese. *Journal of Oral Pathology and Medicine*.

[15] Ranganathan K, Reddy BVR, Kumarasamy N, Solomon S, Viswanathan R, Johnson NW (2000). Oral lesions and conditions associated with human immunodeficiency virus infection in 300 south Indian patients. *Oral Diseases*.

[16] Kerdpon D, Pongsiriwet S, Pangsomboon K (2004). Oral manifestations of HIV infection in relation to clinical and CD4 immunological status in northern and southern Thai patients. *Oral Diseases*.

[17] Adedigba MA, Ogunbodede EO, Jeboda SO, Naidoo S (2008). Patterns of oral manifestation of HIV/AIDS among 225 Nigerian patients. *Oral Diseases*.

[18] Nittayananta W, Chanowanna N, Sripatanakul S, Winn T (2001). Risk factors associated with oral lesions in HIV-infected heterosexual people and intravenous drug users in Thailand. *Journal of Oral Pathology and Medicine*.

[19] Dodd CL, Greenspan D, Katz MH, Westenhouse JL, Feigal DW, Greenspan JS (1991). Oral candidiasis in HIV infection: pseudomembranous and erythematous candidiasis show similar rates of progression to AIDS. *AIDS*.

[20] Nielsen H, Bentsen KD, HØjtved L (1994). Oral candidiasis and immune status of HIV-infected patients. *Journal of Oral Pathology and Medicine*.

[21] Fonseca R, Cardoso AS, Pomarico I (2000). Frequency of oral manifestations in children infected with human immunodeficiency virus. *Quintessence International*.

[22] Barr CE, Torosian JP (1986). Oral manifestations in inpatients with AIDS or AIDS-related complex. *Lancet*.

[23] McCarthy GM, Mackie ID, Koval J, Sandhu HS, Daley TD (1991). Factors associated with increased frequency of HIV-related oral candidiasis. *Journal of Oral Pathology and Medicine*.

[24] Lourenco AG, Figueiredo LT (2008). Oral manifestations in HIV infected patients from Ribeirao Preto, Brazil. *Medicina Oral, Patología Oral y Cirugía Bucal*.

[25] Pedreira EN, Cardoso CL, Barroso Edo C, De Souza Santos JA, Fonseca FP, De Assis Taveira LA (2008). Epidemiological and oral manifestations of HIV-positive patients in a specialized service in Brazil. *Journal of Applied Oral Science*.

[26] Romeu J, Balagué M, Ruiz L (1998). Valve of HIV-1 viral load and CD4 lymphocyte count as determinants of progression to AIDS and survival. *Medicina Clinica*.

[27] Birnbaum W, Hodgson TA, Reichart PA, Sherson W, Nittayannanta SW, Axell TE (2002). Prognostic significance of HIV-associated oral lesions and their relation to therapy. *Oral Diseases*.

[28] Gaitan-Cepeda L, Cashat-Cruz M, Morales-Aguirre JJ (2002). Prevalence of oral lesions in mexican children with perinatally acquired HIV: association with immunologic status, viral load, and gender (AIDS Patient Care and STDs. *AIDS Patient Care and STDs*.

[29] Duggal MS, Abudiak H, Dunn C, Tong HJ, Munyombwe T (2010). Effect of CD4+ lymphocyte count, viral load, and duration of taking anti-retroviral treatment on presence of oral lesions in a sample of South African children with HIV+/AIDS. *European Archives of Paediatric Dentistry*.

[30] Patton LL, McKaig R, Strauss R, Rogers D, Eron JJ (2000). Changing prevalence of oral manifestations of human immunodeficiency virus in the era of protease inhibitor therapy. *Oral Surgery, Oral Medicine, Oral Pathology, Oral Radiology and Endodontics*.

[31] Moura MDG, Grossmann SDMC, Fonseca LMDS, Senna MIB, Mesquita RA (2006). Risk factors for oral hairy leukoplakia in HIV-infected adults of Brazil. *Journal of Oral Pathology and Medicine*.

[32] Eyeson JD, Tenant-Flowers M, Cooper DJ, Johnson NW, Warnakulasuriya KAAS (2002). Oral manifestations of an HIV positive cohort in the era of highly active anti-retroviral therapy (HAART) in South London. *Journal of Oral Pathology and Medicine*.

[33] Ramírez-Amador V, Esquivel-Pedraza L, Sierra-Madero J, Anaya-Saavedra G, González-Ramírez I, Ponce-de-León S (2003). The changing clinical spectrum of human immunodeficiency virus (HIV)-related oral lesions in 1,000 consecutive patients: a 12-year study in a referral center in Mexico. *Medicine*.

[34] Umadevi KMR, Ranganathan K, Pavithra S (2007). Oral lesions among persons with HIV disease with and without highly active anti retroviral therapy in southern India. *Journal of Oral Pathology and Medicine*.

[35] Logan RM, Coates EA, Pierce AM, Wilson DF (2001). A retrospective analysis of oral hairy leukoplakia in South Australia. *Australian Dental Journal*.

[36] Nittayananta W, Talungchit S, Jaruratanasirikul S (2010). Effects of long-term use of HAART on oral health status of HIV-infected subjects. *Journal of Oral Pathology and Medicine*.

[37] Nicolatou-Galitis O, Velegraki A, Paikos S (2004). Effect of PI-HAART on the prevalence of oral lesions in HIV-1 infected patients. A Greek study. *Oral Diseases*.

[38] King MD, Reznik DA, O’Daniels CM, Larsen NM, Osterholt D, Blumberg HM (2002). Human papillomavirus-associated oral warts among human immunodeficiency virus-seropositive patients in the era of highly active antiretroviral therapy: an emerging infection. *Clinical Infectious Diseases*.

[39] Patton LL, Ranganathan K, Naidoo S (2011). Oral lesions, HIV phenotypes, and management of HIV-related disease: workshop 4A. *Advances in dental research*.

